# Raster-scanning optoacoustic mesoscopy biomarkers for atopic dermatitis skin lesions

**DOI:** 10.1016/j.pacs.2023.100513

**Published:** 2023-05-25

**Authors:** T. Nau, C. Schönmann, B. Hindelang, L. Riobo, A. Doll, S. Schneider, L. Englert, H. He, T. Biedermann, U. Darsow, F. Lauffer, V. Ntziachristos, J. Aguirre

**Affiliations:** a Department of Dermatology and Allergology, Technical University of Munich, Munich, Germany; b Chair of Biological Imaging, Technical University of Munich, 81675 Munich, Germany; c Institute of Biological and Medical Imaging, Helmholtz Zentrum Munich, 85764 Neuherberg, Germany; d Munich Institute of Robotics and Machine Intelligence (MIRMI), Technical University of Munich, 81675 Munich, Germany; e Departamento de Tecnología Electrónica y de las Comunicaciones, Universidad Autonoma de Madrid, Madrid, Spain; f Instituto de Investigacion Sanitaria de la Fundacion Jimenez Diaz, Madrid, Spain

**Keywords:** Imaging, Atopic dermatitis, Inflammation, Skin diseases, Eczema, Photoacoustics, Optoacoustics

## Abstract

Atopic dermatitis (AD) is the most common chronic inflammatory skin disease worldwide. Its severity is assessed using scores that rely on visual observation of the affected body surface area, the morphology of the lesions and subjective symptoms, like pruritus or insomnia. Ideally, such scores should be complemented by objective and accurate measurements of disease severity to standardize disease scoring in routine care and clinical trials. Recently, it was shown that raster-scanning optoacoustic mesoscopy (RSOM) can provide detailed three-dimensional images of skin inflammation processes that capture the most relevant features of their pathology. Moreover, precise RSOM biomarkers of inflammation have been identified for psoriasis. However, the objectivity and validity of such biomarkers in repeated measurements have not yet been assessed for AD. Here, we report the results of a study on the repeatability of RSOM inflammation biomarkers in AD to estimate their precision. Optoacoustic imaging analysis revealed morphological inflammation biomarkers with precision well beyond standard clinical severity metrics. Our findings suggest that optoacoustic mesoscopy may be a good choice for quantitative evaluations of AD that are inaccessible by other methods. This could potentially enable the optimization of disease scoring and drug development.

## 1 Introduction

Atopic dermatitis (AD) is the most common chronic inflammatory skin disease worldwide. It is a detrimental disorder that diminishes patients’ quality of life. It is estimated that approximately 20 % of children and 3 % of adults are affected by it. In addition to eczematous lesions, patients suffer from intense pruritus and insomnia, which severely impacts patients’ quality of life [1]. The pathophysiology of AD relies on a type 2 dominant immune response, an impaired epidermal barrier, and a reduced diversity of the skin microbiome [2].

AD is treated step-wise with topical and/or systemic therapies dependent on disease severity. Apart from basic treatment with moisturizers, mild AD is usually treated with class 1–2 topical corticosteroids (TCS) or topical calcineurin inhibitors (TCI). According to the current European guideline, disease flares should be treated with phototherapy and medium to high potency TCS [3]. In case of severe AD, a relapsing disease course or if long-term control cannot be achieved with the aforementioned strategies, the initiation of a systemic therapy is recommended. Apart from unspecific immunosuppressants like methotrexate and cyclosporine, two biologics and three Janus kinase inhibitors (JAKi) have been approved during the last 5 years [4]. Dupilumab, an anti-IL-4/IL-13 receptor antibody, was the first biologic treatment for AD. By blocking key cytokines of type 2 immune responses, it leads to significant improvement of visible signs and subjective symptoms of AD [5]. Tralokinumab is an anti-IL-13 antibody, which also showed significant improvement of Eczema Area and Severity Index (EASI) score in two phase III clinical trials [6]. Furthermore, three JAKi have been approved for the treatment of AD: baricitinib, upadacitinib and abrocitinib. While baricitinib is selective for JAK1/2, upadacitinib and abrocitinib mainly block JAK1 signaling. All three JAKi proved efficacy for the treatment of AD [7]. However, due to current recommendations of the FDA and EMA they should be used with caution in elder patients, patients with cardiovascular comorbidities, smokers and patients with a higher risk for cancer.

Precise assessment of disease severity on a personalized, per-patient basis is required for successful research on new therapeutics and optimization of treatment. It is important to identify ineffective therapies and adjust or change them as early as possible.

Different clinical scores are used to measure the severity of AD, such as EASI [8] and Scoring AD (SCORAD) [9], which are used in combination with the Dermatology Life Quality Index (DLQI) [10]. The DLQI is a patient questionnaire that measures the impact of the disease on the patient’s quality of life. The EASI is based on the intensity of redness, thickness, excoriation, and lichenification as assessed by an experienced rater. SCORAD combines visual observation parameters like the extent of affected skin area, erythema, edema or formation of papules, oozing or crust formation, excoriation, lichenification, and dryness with subjective symptoms such as pruritus and insomnia. Though EASI and SCORAD function as standard outcome measurements in several clinical trials, they are only based on subjective assessments and visual inspection.

Recently, the three-dimensional (3D) mesoscopic imaging technique of ultra-broadband raster-scanning optoacoustic mesoscopy [11], [12], [13] (UB-RSOM), (some authors refer to optoacoustic mesoscopy as acoustic resolution photoacoustic microscopy [14], [15], [16], [17], demonstrated the possibility of offering a label-free precision assessment of skin inflammation severity, with the potential to overcome the drawbacks of visual inspection, including comparison with histological analysis [18]. The UB-RSOM combines pulsed laser excitation and detection of ultrasound waves generated by heat-induced expansion of tissue to detect and visualize optical absorbers (e.g., hemoglobin and melanin when using visible lasers) at sub-10-µm resolution through the whole skin depth [11], [19]. So, UB-RSOM can show several important skin features, such as the epidermis, capillary loops, and even the dermal vasculature with high resolution (sub-10-µm).

The unique combination of contrast, resolution, and penetration depth of UB-RSOM allows the creation of highly detailed pictures of the skin microvascular structure and thus generates useful quantitative biomarkers of inflammation obtained from image analysis, since the pathology of skin inflammation is strongly linked to drastic changes in the microvascular architecture of the skin [18], [20]. Pure ultrasound and optical technologies like reflection confocal microscopy, optical coherence tomography, and multiphoton optical microscopy lack the penetration depth or contrast to comprehensively characterize the microvascular structure [16] and fail to provide severity assessment metrics. Central features to achieve a comprehensive view of the microvascular structure are the ultrabroadband features of the UB-RSOM transducers (detected frequencies from 10 MHz to 120 MHz), which enable the detection of the wide range of optoacoustic signals (frequency-wise) generated in the skin microvascular structure [18].

UB-RSOM inflammation biomarkers have been mainly studied in psoriasis [18], [20]. In the most recent study, their precision was quantified by performing a repeatability study with psoriasis patients [20]. More specifically, it was shown that the fine quantitative biomarkers obtained by the UB-RSOM were minimally affected by noise, motion, or errors introduced by the device operator during data collection. An important milestone was showing that the variable pressure exerted by the UB-RSOM scan head on psoriatic plaques did not compress the dermis and corrupt biomarker calculations.

However, these biomarkers have not been studied in detail in the context of AD. It is not clear whether the image features and parameters that can be measured from the images of psoriatic skin can be observed and measured in eczematous skin. Moreover, the compressibility of psoriasis plaques is not the same as that of eczematous skin, owing to their different clinical presentations. Therefore, it is not clear if the pressure of the scan head on the skin will affect the precision of such parameters, which would need to be determined.

Here, we hypothesized that despite the difference in clinical presentation between AD and psoriasis, the UB-RSOM biomarkers obtained for psoriasis [20], [21] can be measured in eczema with similar repeatability.

To confirm our hypothesis, we imaged a large set of skin locations with eczema (n = 10) corresponding to different patients using UB-RSOM. The measurements allowed us to compare the eczema images with standard RSOM images of psoriatic plaques to elucidate whether psoriasis biomarkers can be observed in eczema. Furthermore, we examined the repeatability of the biomarker calculations. This research work paves the way toward establishing UB-RSOM as a precision alternative to the low performance of visual indexes for severity assessment of eczema, with implications for disease scoring and drug discovery.

## 2 Methods

### 2.1 The RSOM system, image acquisition, and image reconstruction

**Fig. 1:**
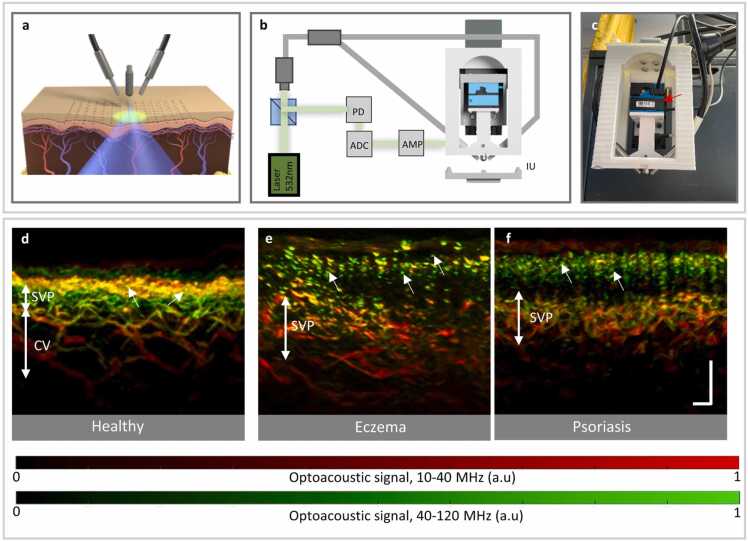
Imaging of eczema using UB-RSO. a. Schematic of the UB-RSOM experimental arrangement, consisting of two fiber bundles for illumination and a transducer that is raster-scanned parallel to the skin surface and acquires optoacoustic signals (A lines). b. Schematic of the UB-RSOM system. A 2 ns laser pulse at 532 nm is generated and directed to the sample by optical fiber bundles. A small part of the beam is diverged using a beam splitter to a PD, which triggers the ADC. The optoacoustic signals collected by the transducer are amplified and sent to the ADC. The transducer is placed in the scanning head. An interface unit (IU) is responsible for proper attachment to the skin. c. Photograph of the UB-RSOM scanning head and articulated arm. The transducer is attached to two motorized stages for scanning along the x- and y-axes (red arrow), together with a third motor that allows adjustment of the height of the transducer in the z-direction. d. UB-RSOM cross-sectional image of healthy skin showing the clearly resolved vessels in the dermis, which are distributed in two structures: the SVP and the CV. The tips of the capillary loops are also visible (white short arrows). The loops appear interleaved with the epidermal structures since they extend slightly toward the surface through the dermal rete ridges. e. UB-RSOM cross-sectional image of eczematous skin, showing the top part of elongated capillary loops (white short arrows) that almost reach the skin surface through elongated rete ridges. The capillaries appear in green, interleaved with widened epidermal structures that result from acanthosis. Such epidermal structures appear red with poor contrast due to loss of pigmentation. Below the epidermis, a dilated and dense vascular structure in the dermis is visible. Such vascular structures correspond to the widened SVP. f. UB-RSOM cross-sectional image of psoriatic skin also show the elongated capillary loops and the widened SVP. Scale bars, 250 µm. RSOM, raster-scan optoacoustic mesoscopy; ADC, analog-to-digital convertor; PD, photodiode; SVP, subepidermal vascular plexus; CV, connecting vessels.

The UB-RSOM apparatus is a custom-made system (Fig. 1a–c). It uses a 532 nm Nd:YaG laser (Wedge HB.532, Bright-solutions SRL, Pavia, Italy) with a pulse length of 0.9 nanoseconds for excitation. The repetition rate was set to 500 Hz, ensuring that the light energy delivered to the skin surface was below 3.75 µJ/mm [2], as indicated by the American National Standards Institute. The illumination spot was a disc of 5 mm diameter and the per pulse energy 35 μJ. The ultrasound detector is a spherically focused piezoelectric transducer (frequency range 10–120 MHz) with a central frequency of 55 MHz (Sonaxis, Besancon, France) and it is integrated in a scan head together with the illumination bundles. More specifically, the transducer was attached to three motorized stages (x, y and z, respectively). The x-y stages are small, having a size of 35 mm × 35 mm × 15 mm (Physik Instrumente, Karlsruhe, Germany) and are used to scan the transducer and bundles along the acquisition grid. The z stage is the MTS50-Z8 from Thorlabs (Newton, USA) and allows to place the focal point of the transducer slightly above the skin surface (∼ 300 µm). The acquisition grid covers a 4 × 2 mm field of view and the acquisition step is 20 µm yielding an acquisition time of ∼ 70 s.

An interchangeable interface unit is placed on top of the skin area to be imaged. Such interface unit has an optically and acoustically transparent plastic membrane. After placement, the unit is filled with 1.5 ml of water in order to couple acoustically the transducer with skin area. Once the unit is filled, the scan head is attached to it.

The procedure for placement of the system on a specific skin area was guided by the ink fiducial marker procedure previously described [20]. This procedure allows the imaging of the same skin area in longitudinal studies for the assessment of therapeutic effects. The procedure consists of defining a region of interest (ROI) by marking two green ink dots in the skin separated by a gap of 4 mm, which corresponds to the UB-RSOM’s transversal field of view. For each imaging session, the scan head was positioned with care, ensuring that the ink dots were placed at the limits of the field of view and the center of its coronal axis [20].

Once the scan head is in place the data acquisition is launched. The raw data is processed as follows: first the signals are split in two frequency bands (10–42 MHz and 42–120 MHz) by means of a 4th order Butterworth filter. From the filtered data two images are reconstructed using the so-called universal back projection algorithm [22] (each image corresponds to a frequency band). In order to represent the reconstructed data a red, green, and blue (RGB) image is used. The low-frequency band reconstruction occupies the red channel and the high-frequency reconstruction occupies the green channel, allowing to discriminate tissue components depending on their size. The smallest venules and arterioles appear in green, while the larger vessels of microvascular plexus appear in red. The melanin layer appears mainly in red tones, which allows to differentiate the capillary loops [18]. The low and high frequency components are equalized as previously described [18], in order to mitigate the preferential absorption by tissue of the high frequency components. Further technical details of the system are available elsewhere [18], [20]. The RSOM employs motion correction algorithms to compensate for slight involuntary movements of a patient’s body [21].

The resolution of the system is ∼ 30 µm (lateral) and ∼ 7 µm (axial) up to ∼ 1.2 mm deep. Further degradation is expected beyond these depths due to acoustic absorption. In [18] the resolution of the system is studied in detail. It is well established that up between 1.2 mm and 2.5 mm the resolution degrades only by 10 µm [18].

### 2.2 The pathology of eczematous skin according to RSOM

As a first step towards confirming that the UB-RSOM biomarkers previously defined for psoriasis [20] can be obtained in AD, we made a comparison between a representative UB-RSOM image of healthy skin, psoriatic skin, and eczematous skin to identify common pathological features of inflammation, including capillary loop elongation and dilatation, acanthosis, and expansion of the sub-epidermal vascular plexus. Our previous studies showed that if such features are displayed in the images, the biomarkers “Mean Capillary Loop Length” (MCLL) and “Mean Plexus Width” (MPW) can be measured.

The sex of each subject was masculine, and the ages were 27 for healthy skin, 30 for eczematous skin, and 42 for psoriatic skin. The study protocol was approved by the ethics committee of the Faculty of Medicine at the Technical University of Munich.

Moreover, we examined the images from the repeatability study (see below) to further demonstrate the compatibility of MCLL and MPW for the eczema case.

### 2.3 Repeatability study

Once we demonstrated that the biomarkers previously derived from psoriasis images could be assessed in AD, we performed a repeatability study. The experimental protocol was approved by the Ethics Committee of the Faculty of Medicine at the Technical University of Munich. A total of 10 locations in eczema skin with a variety of clinical presentations were measured twice in 5 patients (3 women and 2 men, aged 21–85, mean age 43 years, 2 skin locations per patient) who were enrolled in the study after providing written informed consent. Therefore, the number of samples is 10 (n = 10) and we performed 20 measurements. All the RSOM images are shown in the Supplementary data. All participants were patients of the Department of Dermatology at the University Hospital of the Technical University of Munich who had been diagnosed with AD. The repeatability study was designed following the guidelines provided in the study by Bartlett and Frost [23] For each skin location, two measurements of the same eczematous skin area were recorded 10 min apart. Once the first measurement was performed, the scan head was completely detached from the patient skin. Between measurements, the patients were free to move or walk. The MCLL and MPW parameters were derived for each measurement by an expert operator using the procedure described below. The repeatability coefficient was calculated as $1.96*\sqrt{2}*\mathrm{std}$ where “std” is the within-subject standard deviation and was calculated using a one-way analysis of variance (ANOVA) using Matlab, assuming normality in the error distribution. Normality was checked using the Anderson-Darling test and a 95 % confidence interval was calculated [23] using the following expression [23]:

$$\left(\sqrt{\frac{\mathrm{SSW}}{\chi_{n,0.975}^{2}}},\sqrt{\frac{\mathrm{SSW}}{\chi_{n,0.275}^{2}}}\right)$$

where $\chi_{n,p}^{2}$ is 100 × pth centile of the Chi-square distribution with n degrees of freedom (in here, n is the number of measured skin sites) and SSW is the within-subject sums of squares from the one-way ANOVA table.

### 2.4 Calculation of biomarkers

The procedure for the calculation of MCLL and MPW can be found elsewhere [20] and is reproduced here for completeness.

Once images were acquired and reconstructed, the first step in obtaining the biomarkers resolved by UB-RSOM was to flatten the skin surface in the reconstructed images. This step was performed manually as follows: the entire 3D image was divided into 20 µm thick slices in the y-direction. For each slice, the maximum intensity projection (MIP) along the y-direction was calculated. In MIPs, the skin surface can be observed univocally (provided in the Results section). For every MIP, the operator placed ten points along the skin surface. Using a spine-based interpolation algorithm, whole surfaces were extracted and the skin was flattened (details on the surface extraction method can be found elsewhere [20]. The MIP image along the y-axis was subsequently computed from the flattened skin images. The MCLL and MPW were calculated manually from the MIP (more details in results).

## 3 Results

The application of UB-RSOM to healthy skin (Fig. 1d), eczematous skin (Fig. 1e), and psoriatic skin allowed a side-by-side comparison of its performance in resolving the relevant pathological dermal features for precision biomarker calculations. In healthy skin, the UB-RSOM reveals the epidermis and underlying dermal structures. Specifically, the melanin and capillary loops (CL) are clearly resolved in the epidermis, whereas the subepidermal vascular plexus (SVP) and connecting vessels (CV) are visible in the dermal layer (DR). In green (high frequency band), we can observe the tip of the capillary loops and the smallest vessel of the upper plexus, while the larger vessels appear in red. The capillary loop tips can be observed and discriminated from the melanin layer. This general appearance changes in eczematous skin, wherein UB-RSOM captures the underlying acanthosis, drastic elongation and dilation of the capillary loops, and expansion of the sub-epidermal vascular plexus. The same features were also observed in psoriatic skin (Fig. 1f). Hence, MPW and MCLL can be calculated from the UB-RSOM images of the AD.

**Fig. 2:**
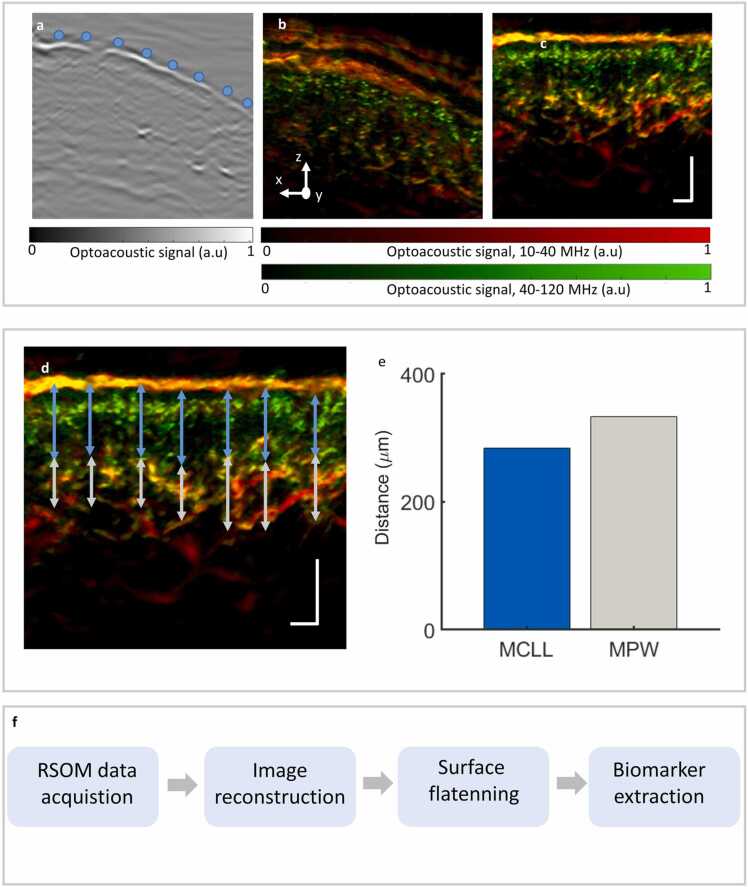
Method for computing biomarkers in reference to the skin surface. a. From a 20 µm thick MIP several points on of the skin’s surface are selected manually (blue spheres). b. UB-RSOM cross sectional view prior to surface flattening. c. UB-RSOM cross sectional view after surface flattening. d. The mean of the length of the blue arrows corresponds to the MCLL, whereas the mean of the length of the red lines corresponds to the MPW. e. The MCLL and MPW values that correspond to d. f. Steps for biomarker extraction. Scale bars, 250 µm. RSOM, raster-scan optoacoustic mesoscopy; MCLL, mean capillary loop length; MPW, mean plexus width.

In Fig. 2, we show the process of extracting RSOM biomarkers from the reconstructed data of a representative patient with AD. The surface-flattening procedure is shown in Fig. 2a–c. Fig. 2a shows a 20 µm thick MIP and 10 surface points in the y-direction used to interpolate the surface. In Figs. 2b and 2c, we show the effects of flattening on the reconstructed images.

Fig. 2d shows the methodology for biomarker calculation (MCLL and MPW). In order to compute the MCLL a segment is placed in the UB-RSOM lateral view. The segment is oriented in the z-direction and connects the upper edge of the microvascular plexus with the tip of the capillary loops closest to the skin surface. This process is performed seven times along the x-axis (blue arrows in Fig. 2d). At each time the length of the segment is computed and the mean value calculated. A similar operation was performed to calculate the MPW. In the MPW case, the segment now covers the entire vascular plexus (white arrows in Fig. 2d). The results of these calculations are shown in Fig. 2e. Fig. 2f display each of the steps of the process.

**Fig. 3:**
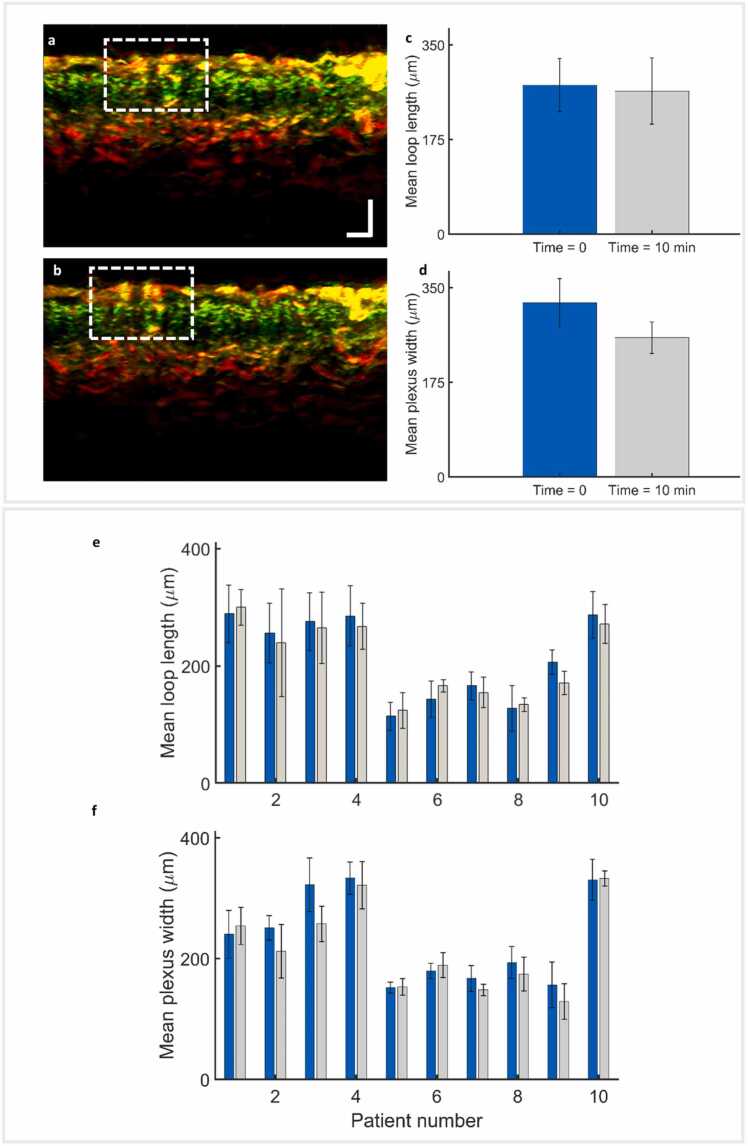
Repeatability of biomarkers extracted from UB-RSOM measurements. a–b. Sample images for one skin location from the repeatability study. An initial image was recorded (t = 0), followed by a second image after 10 min (t = 10). The dashed rectangle shows the same structure in both images. It can be observed that the scan head could not be placed at exactly the identical position in both measurements. The positioning error is about 300 µm. c and d. Values of the MCLL and MPW corresponding to images (a) and (b). The measurement could be repeated with a difference of a few tens of micrometres. d. Values of the MCLL calculated from both measurements (t = 0 and t = 10 min) for each skin location. e. Values of the MPW are calculated from both measurements (t = 0 and t = 10 min) for each skin location. Scale bars: 250 µm. RSOM, raster-scan optoacoustic mesoscopy; MCLL, mean capillary loop length; MPW, mean plexus width.

Fig. 3 presents the results of the repeatability study. Figs. 3a and 3b show the images of the repeatability study corresponding to the first skin location of patient 1. The reconstructed RSOM image obtained at t = 0 is shown in Fig. 3a, and the reconstruction corresponding to t = 10 min is shown in Fig. 3b. A comparison between t = 0 and t = 10 min reveals that the scan-head placement method based on ink fiducial makers [20] can lead to small discrepancies of a few hundred microns in the positioning.

Regarding the calculation the biomarkers, for the MCLL we obtained 275 µm and 265 µm for t = 0 min and t = 10 min, respectively (Fig. 3c), whereas, for the MPW, we obtained 322 µm and 257 µm (Fig. 3d). All biomarker values obtained in this study are displayed in Figs. 3e (MCLL) and 3f (MPW). We obtained a repeatability coefficient of 34.6 µm for MCLL and 54.1 µm for MPW (the 95 % confidence interval is 24.2–60.7 µm for the MCLL and 37.7–94.8 µm for the MPW). The low repeatability coefficients show that possible intra-operator errors like positioning, variable pressure on the skin, motion, etc. barely affect biomarker measurements.

## 4 Discussion

In this study, we confirmed that UB-RSOM skin inflammation biomarkers previously derived for psoriasis can also be obtained from images of AD. Moreover, for the first time, we performed a medium-sized repeatability study. Our results revealed that the repeatability coefficient for MCLL is 34.6 µm and for MPW 54.1 µm in AD, which indicates that a difference greater than these values, when measured longitudinally in future studies, can be attributed to an increase or decrease in the severity of the disease. So, MCLL and MPW might be useful as objective and accurate biomarkers of the severity of AD, overcoming the drawbacks of standard severity metrics such as SCORAD or EASI.

The values obtained for the repeatability coefficient are similar to the values obtained for psoriasis in the case for MCLL (28 µm in the psoriasis case). However, the plexus width values are worse MPW (29 µm in psoriasis). Also, the 95 % confidence intervals are 24.2–60.7 µm for the MCLL and 37.7–94.8 µm for the MPW. The worse performance of the MPW could be due to the structure of the vascular plexus in AD, which seems to be less dense than the plexus in psoriasis, and therefore, finding the boundaries of the plexus is difficult. Further repeatability studies will allow to narrow down the confidence intervals and also determine possible bias effects for large biomarker values. Also, further work should be focused on the ability of RSOM to discriminate eczema from psoriatic lesions.

Other authors have proposed objective alternatives to the SCORAD and EASI indexes [24] based on computational imaging methods for measuring the extension of the disease and assessing the epidermal barrier using bioengineering methods [24]. Such parameters related to the skin surface cannot be compared directly to RSOM biomarkers, which are measured deep in the skin. Future studies should compare the sensitivity of both techniques for follow-up treatment.

As imaging techniques become increasingly interesting in the field of dermatology, several other systems have been proposed in the literature. In 2020, Ho et al. [25] published an article on confocal Raman spectroscopy (CRS) for the assessment of the skin barrier function in AD. Therefore, the authors aimed to stratify the disease severity. However, the CRS system only features a depth of 100 µm, whereas the RSOM system has a depth of 1.5 mm (green light) and 5 mm (infrared light). Ho et al. focused on the concentration of moisturizing factors in the skin, which is one of the factors that play a role in the pathophysiology of AD. In contrast, RSOM measures the thickness of the skin resulting from inflammation typical of AD. Therefore, we analyzed a combination of pathogenic factors and their results, which is why RSOM can be directly used in daily clinics or routines.

In a different approach, an alternative set of RSOM biomarkers was defined and automatically obtained from the reconstructed images [26], [27]. In this approximation, the epidermis thickness, total blood volume, vessel diameter in the dermis, and the ratio between low- and high-frequency signals (LHFR) can be extracted from the images. The epidermis thickness may be related to MCLL, since acanthosis generally implies an increase in the length of the capillary loops. However, no repeatability studies have been reported for such biomarkers. Similarly, total blood volume, vessel diameter, and LHFR are parameters that are strongly related to angiogenesis and therefore should display some degree of correlation with MPW. Again, no repeatability studies have been conducted. Therefore, further work should compare both manual and automatic approaches in terms of precision.

In line with the findings of previous studies, we observed that under the light of RSOM, psoriasis, and AD display similar features related to the microvascular structure [18]. Such similarities allow the use of the same biomarkers for both diseases. Further work should be carried out to characterize the ability of the biomarkers for AD treatment monitoring in order to better assess the clinical advantages that they bring over visual indices. In particular, we expect that the high precision of the MPW and MCLL will allow the observation of therapeutic effects of treatment that, with current severity assessment methods, remain subclinical. This ability has been demonstrated in psoriasis [18]. The performance for extracting AD biomarkers of dual optoacoustic approaches that combine optical resolution optoacoustic microscopy with acoustic resolution photoacoustic tomography should be also considered [28], [29].

In contrast to the established scores UB-RSOM can only be used for local severity assessment. The recent development of fast RSOM, with an acquisition times of ∼ 15 s for a FOV similar as the one used for acquiring the data presented here, opens the door for multiple-site imaging within an acceptable time. Future steps should include performing longitudinal studies of patients undergoing treatment to assess the sensitivity of the UB-RSOM biomarkers against the sensitivity of the SCORAD and EASI indices. In order to be adopted in the clinic, the general usability of RSOM has to be improved. Details like the use of water, the manual surface extraction method and alike represent a usability challenge that has to be overcome.

## 5 Conclusion

The high precision and accuracy of the MPW and MCLL biomarkers suggest that UB-RSOM can be an alternative to SCORAD and EASI indices for severity assessment, with the potential to catalyze disease scoring and drug discovery in AD. The biomarkers presented may not change clinical practice right away, but their abilities emphasize their potential to improve clinical research and, subsequently, clinical routines in patient care. Their calculation is easy to learn, can quickly be done and will provide an objective impression of the extent of inflammation in the skin. The biomarkers’ numerical values can easily be compared and give an impression of a lesion’s depth extent and are therefore superior to subjective tools like SCORAD or EASI.

## Declaration of Competing Interest

The authors declare the following financial interests/personal relationships which may be considered as potential competing interests: Vasilis Ntziachristos reports a relationship with Ithera Medical that includes: equity or stocks.

## Data Availability

Data will be made available upon reasonable request.Patient data might be subjected to confidentiality.
