# Supplementary material for: Raster-scanning optoacoustic mesoscopy biomarkers for atopic dermatitis skin lesions
Source: Photoacoustics. 2023 May 25;31:100513. doi: 10.1016/j.pacs.2023.100513 (PMC10236218; doi:10.1016/j.pacs.2023.100513)
Supplement: Supplementary file 1 — Supplementary material [file mmc1.docx]

**Raster-­scanning ­optoacoustic­ mesoscopy biomarkers for atopic dermatitis skin lesions.**

**Authors**

T. Nau et al.

**Supplementary data.**

In supplementary figure 1 we show all the RSOM images obtained for the repeatability study.


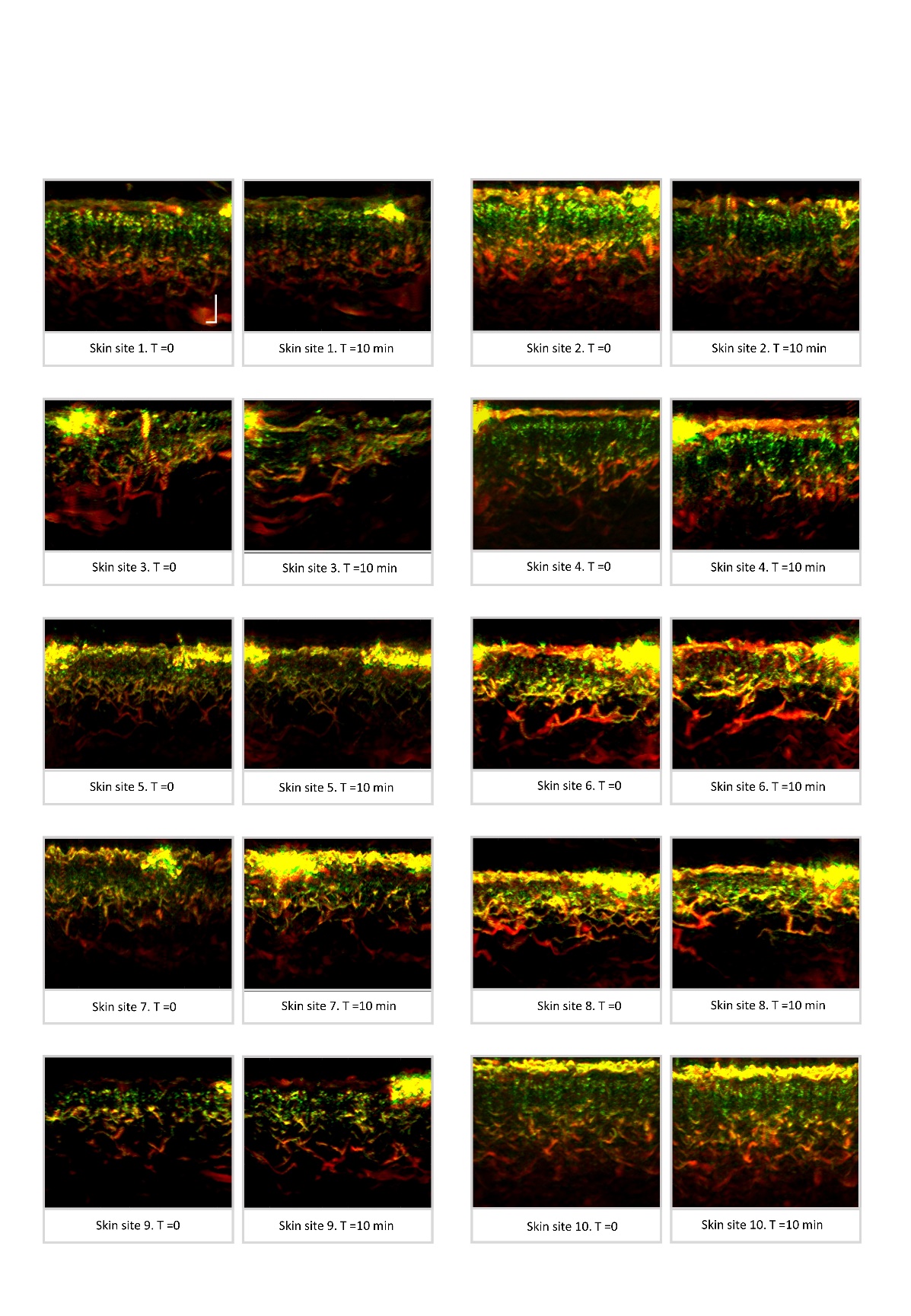


Supplementary figure 1. All the images obtained for the repeatability study at T=0 and ten minutes after (T=10 min).
